# Examining the Associations between Adverse Childhood Experiences, Health Risk Behaviours, and Psychological Well-Being in a Convenience Sample of Lithuanian University Students

**DOI:** 10.3390/ijerph19063253

**Published:** 2022-03-10

**Authors:** Ilona Laurinaitytė, Luciana C. Assini-Meytin, Ksenija Čunichina

**Affiliations:** 1 Institute of Psychology, Faculty of Philosophy, Vilnius University, 01513 Vilnius, Lithuania; ksenija.cunichina@fsf.vu.lt; 2 Mental Health Faculty, Johns Hopkins Bloomberg School of Public Health, Baltimore, MD 21205, USA; lassini@jhu.edu

**Keywords:** adverse childhood experiences, health risk behaviour, psychological well-being, university students

## Abstract

This study examines the associations between adverse childhood experiences (ACEs), health risk behaviours, and psychological well-being among Lithuanian university students. A cross-sectional survey was carried out with a convenience sample of 393 students (80.7% females and 19.3% males) recruited from mostly undergraduate courses (96.4%) in Lithuanian universities. Participants, aged 18–25 years (21.07 ± 1.53), completed a web-based survey in which they were asked to retrospectively self-report on ACEs while answering questions on health risk behaviours (e.g., smoking, substance use, riding a car with a drunk driver) and psychological well-being. Only 8.7% of the study sample experienced no ACEs, and almost half of the sample (48.9%) experienced ≥4 ACEs. Findings from adjusted models showed that, compared with students with no ACEs, those who experienced ≥4 ACEs had higher odds of lifetime illicit drug use (AOR = 2.73, *p* < 0.05), riding with a drunk driver (AOR = 2.44, *p* < 0.05), suicidal ideation before age 18 (AOR = 28.49, *p* < 0.01) and in the past 12 months (AOR = 5.39, *p* < 0.01). An increased number of ACEs was also associated with lower psychological well-being (*B* = −3.94, *p* < 0.001). Findings from this study have implications for mental health professionals as well as university administrators, as students with a higher number of traumatic experiences may require greater levels of support and services.

## 1. Introduction

Exposure to adverse childhood experiences (ACEs), such as different types of abuse, parental substance misuse, or parental imprisonment, is a significant public health problem [1,2,3]. Numerous studies have demonstrated the negative impact of ACEs on mental and physical health outcomes and well-being throughout life [4,5,6,7]. An increased number of reported ACEs has also been associated with more serious negative health outcomes and multiple health risk behaviours, including substance abuse, chronic conditions, psychiatric disorders, and attempted suicide [8,9,10,11,12]. More recently, Figueiredo-Braga et al. [13] noted that people exposed to ACEs seem to be more prone to adjustment difficulties when facing the extreme stress and health-related risk of the COVID-19 pandemic.

Every year, over half of children around the world face various forms of violence [14]. Nonetheless, the prevalence of ACEs worldwide is unknown [15]. While most ACE studies have been conducted in high-income countries (see, e.g., [16]), there is evidence that the prevalence of ACEs is higher in countries with lower per capita incomes [2,17]. Moreover, the number of ACEs may vary within the same world region, for example, in Europe. According to the World Health Organisation (WHO), the prevalence of childhood adversity appears to be higher in eastern European countries than in their western counterparts [17]. Although research on childhood traumatic experiences is growing, to our knowledge, only a few studies have been conducted in central and eastern Europe, especially in the Baltic countries [18,19,20]. For instance, Bellis et al. [18] conducted a study in eight eastern European countries (*n* = 10,696) and found that 18.6% of respondents reported experiencing physical abuse in childhood and 7.5% reported experiencing sexual abuse (5.7% for males and 13.4% for females). Another study based on a sample of 1299 Lithuanian adolescents (mean age 14.24, range 12–16 years) measured lifetime abuse exposure (e.g., physical abuse, emotional abuse, neglect, online sexual violence, sexual abuse from adult and sexual abuse from peers) and found that 71% of participants reported at least one type of abuse [20].

A health risk behaviour can be described as an activity carried out by an individual that increases the probability of adverse health outcomes [21]. Numerous studies have shown that health risk behaviours such as substance abuse, risky sexual behaviour, and suicidal ideation and attempts are associated with an increased number of ACEs [4,15,22,23,24], which may contribute to the global burden of disease. Globally, alcohol, illicit drug, and tobacco use are well-documented main risk factors for premature death and disability [25,26]. For instance, alcohol use was the leading risk factor for deaths worldwide among the age group 15–49 years in 2016 [27]. In addition, European countries had the highest prevalence of daily tobacco use and heavy episodic alcohol use with the highest prevalence of daily tobacco use and heavy episodic alcohol use [26]. Based on the Organisation for Economic Cooperation and Development report (OECD, 2019), the alcohol consumption rate in Lithuania is higher than in any other EU country (exceeding the EU average by 25%) [28]. Moreover, around 25% of all road deaths in the EU are related to alcohol [29].

Impaired driving and suicide are also significant public health problems associated with ACEs. Motor vehicle collisions were found to be the leading cause of death for young people in several countries, and alcohol had a significant impact on the fatality rate [30,31]. In recent years, researchers studying risky behaviour related to driving started to draw more attention to the characteristics of both drivers impaired by alcohol and passengers who were riding with them [32]. Being young was found to be a risk factor for travelling with drunk (adult or peer) drivers [30,32]. As for suicide, Lithuania also has persistently the highest rates of suicide in Europe, especially among the male population [28,33]. Suicide is one of the main external causes of death for young people aged 14–29 years in Lithuania [34]. Notably, suicide rates for adolescents aged 15–19 years in Lithuania are much higher than in other EU countries [35].

Adverse childhood events are also strongly correlated with a general lack of psychological well-being. Psychological well-being encompasses various subjective, social, and psychological aspects, as well as health-related behaviours and practices, that are meaningful to an individual and allow them to fully function [36,37,38]. According to Ryff [38], there are positive associations between psychological well-being and individual health as well as general life satisfaction. Conversely, negative life experiences such as ACEs were found to be related to lower psychological well-being and greater mental health problems [7,39].

Most research on ACEs has been conducted in the general adult population or in samples of vulnerable children or adults, but information on the association between ACEs and outcomes in university students is currently missing [40,41]. Further, Browning et al. [42] noted that university students are increasingly recognised as a vulnerable population that suffers from higher levels of substance abuse, depression, other health risk outcomes and is most strongly affected by COVID-19. Thus, this study aimed to examine the associations between adverse childhood experiences, health risk behaviours, and psychological well-being among Lithuanian university students. It was hypothesised that an increase in the number of ACEs would be associated with increased health risk behaviours and lower psychological well-being.

## 2. Materials and Methods

### 2.1. Participants

A cross-sectional survey was carried out with a convenience sample of 393 students that consisted of 80.7% females (*n* = 317) and 19.3% males (*n* = 76). Students were recruited from mainly undergraduate courses (96.4%) at Lithuanian universities. The majority of students (90.6%) were from two of the largest universities in Lithuania–Vilnius University and Vytautas Magnus University. The remaining participants were from other eight Lithuanian universities. Participants were ranging in age from 18 to 25 years (*M* = 21.07, *SD* = 1.53). 

### 2.2. Measures

Sociodemographics included the participant’s age, biological sex, educational institution, study program, study year, highest parental education (separately for mother and father), living situation, and whether or not they possessed a driver’s license.

Adverse Childhood Experiences categories were mainly adopted from earlier ACEs studies [1,43]. The authors also included other ACEs categories that were informed by previous research (e.g., parental death [2]) and were pertinent to the life of Lithuanian youth (e.g., parental migration). As a result, 13 categories of adverse childhood experiences (related to participants’ first 18 years of life) in total were coded dichotomously (yes/no): emotional abuse (if caregivers cursed, humiliated, or threatened to physically hurt participant), physical abuse (if caregivers pushed, grabbed, hit participant), sexual abuse (if sexual acts were perpetrated against the participant by any adult or other minor at least five years older), substance abuse in household (participant lived with someone who abused drugs or alcohol), incarceration in household, mental illness in household (participant lived with someone who had depression or tried to commit suicide), witnessing domestic violence, parental separation or divorce, parental migration (caregiver lived away in other country for six months or longer), death of parent, placement in foster home, emotional neglect (participant felt that no one in family consider them important or loved them) and neglect (participant very often had nothing to eat, wore dirty clothes, and parents were too intoxicated to take care of them). ACE scores were calculated by adding the number of questions to which participants responded that they had experienced the event (range 0–13). It is important to note that childhood emotional abuse, physical abuse, or neglect was coded as present if participants mentioned that they experienced those events at least once [41]. The categories of ACE scores were also categorised as none, one, two, or three and more (≥4). 

Health risk behaviours were assessed using a questionnaire adopted from Bulotaitė (2014) [44]. Questions about health risk behaviour included smoking (tobacco and/or electronic cigarettes), consumption of alcohol, illicit drugs, and prescribed drugs (for intoxication purposes, without a medical prescription), suicide ideation, risky driving, and risky sexual behaviour. Participants were asked if they have ever smoked cigarettes, used alcohol, illicit drugs, prescribed drugs, and had sexual intercourse. A positive answer was followed by the question regarding their age when they had the first experience (except for prescription drug abuse). Items were assessed in the past 12 months and in the past 30 days (which corresponded to the period of the first lockdown implemented during the COVID-19 pandemic in Lithuania). The risky driving experience assessment included two categories: ever driving after alcohol consumption and riding with a drunk driver (assessed as lifetime, in the past 12 months, and in the past 30 days). Participants were also asked about having suicidal ideation before their 18th birthday and in the past 12 months.

Psychological well-being was evaluated by using the Lithuanian Psychological Well-Being Scale (LPGS-P; Bagdonas et al., 2013) [36]. The scale includes 17 items disclosing satisfaction with 17 areas of well-being: household conditions, financial situation, work, studies, family, daily life, personality (self), status in society, interpersonal relationships, physical health, mental health, leisure time, gender identity, past life, current life, future and life in general. The items were evaluated on an 11-point Likert type scale from zero to ten. The total score of 17 items was calculated. Higher scores indicate greater psychological well-being. The LPGS-P scale demonstrated excellent internal consistency in this sample (Cronbach’s *α* = 0.91).

### 2.3. Procedure

Participants completed a web-based survey instrument with items on retrospective reports of ACEs, health-risk behaviour outcomes, and psychological well-being. Students were recruited from Lithuanian institutions of higher education via emails sent out by university liaisons (e.g., deans of education, study program directors, professors) and student organisations. University students aged 18 years or older and enrolled in any study program were eligible to participate in the study. Participation was strictly voluntary, and no course credit was provided for participation. Data were collected during the first national lockdown wave of COVID-19 between April and May 2020. Consequently, a survey was created, and data were gathered using an online platform (Google Forms) only. The study was approved by the Ethics Committee for Psychological Research of Vilnius University (Lithuania), 27 February 2020, Nr. 38.

### 2.4. Statistical Analysis

Sample descriptive statistics were estimated with univariate analysis to determine the proportions and means across participants’ demographic characteristics, ACE types, cumulative number of ACEs, and ACE scores. Bivariate analyses (*t*-test for continuous variables and chi-square for categorical variables) were used to estimate biological sex differences across participants’ demographic characteristics, ACE types, the cumulative number of ACEs, and ACE scores. Next, one-way ANOVA (for continuous variables) or chi-square tests (for categorical variables) were used to estimate the associations between the cumulative number of ACEs and outcomes (i.e., health risk behaviours and psychological well-being). In order to decrease the potential for family-related errors, a Bonferroni correction was used. The alpha level for each external criterion was corrected by dividing it by the number of tests (i.e., *α* = 0.05/6 = 0.008). Finally, separate multivariable regression models were used to estimate the association between health risk behaviours, psychological well-being, and ACEs, controlling for demographic characteristics (i.e., age and biological sex). Multivariable logistics regression models were used for binary outcomes (i.e., health risk behaviour outcomes), and linear regression was used for a continuous variable as an outcome (i.e., psychological well-being). Notably, for an improved model fit, we used a categorical measure of ACEs (i.e., none, one, two or three and ≥4) as the independent variable in logistic regression models; we used a continuous measure of ACEs (i.e., ACE score) as an independent variable in the linear regression model. We assessed the appropriateness of our logistic regression models based on the following parameters: Wald statistics *p* < 0.05; classification > 50%; Cook’s distance ≤ 1 and Hosmer–Lemeshow test *p* ≥ 0.05. All our logistic regression models met these parameters. All analyses were conducted using IBM SPSS 25.0 software package.

## 3. Results

Participants’ demographic characteristics and ACE percentages and means are presented in Table 1. Participants were on average 21 years old (age range 18–25) and mostly from social sciences and humanities programs (80.5%). Parents of study participants were well educated; approximately three-fourths of participants (76.4%) had a mother with a college or university level education, and more than half of them (62.6%) had a father with a college or university level education. Participants’ living situation was distributed among living with parents (34.4%), in a campus dormitory (24.3%), or in a rented apartment either alone or with others (33.1%). Almost two out of three participants (64.9%) possessed driver’s licences. As for ACE types, a significant proportion of participants reported emotional abuse (68.4%) and emotional neglect (64.9%) and almost half experienced physical abuse (48.9%). As for ACE categories, only 8.7% of the study sample experienced no ACEs, and almost half of the sample (48.9%) experienced ≥4 ACEs. Participants experienced 3.36 ACEs on average (range 0–10).

Study results revealed that a vast majority of the sample (93.4%) had at least one substance use lifetime experience (tobacco, alcohol, illicit drugs, or prescribed drug abuse). The distribution of health risk behaviour outcomes and psychological well-being scores by ACE score categories are presented in Table 2. On average, participants were 16.3 years old (*SD* = 0.10) when first tried alcohol and 16.9 years old (*SD* = 2.11) when they started smoking. Participants’ mean age of first use illicit drug use and first sexual intercourse was 18 (*SD* = 0.14 and *SD* = 1.92, accordingly). In this sample, 10.9% of the students reported driving after alcohol consumption (which consisted of 16.9% of those who possess a driver’s licence). Results showed a statistically significant (*p* < 0.008) binary-graded association between the cumulative number of ACEs and several outcomes regarding health risk behaviours, including use of illicit drugs during lifetime and in the past 30 days, ever riding with a drunk driver, suicidal ideation before age 18, and suicidal ideation in the past 12 months (Table 2). Additionally, as shown in Table 2, results revealed a strong, graded association between the ACE score categories and psychological well-being scores (*p* < 0.001), since, by increasing the cumulative number of ACEs, participants experienced lower levels of psychological well-being.

After controlling for demographic characteristics (age and biological sex), results from logistic regression models for adverse childhood experience exposure and health risk behaviours show that students in the group with the highest cumulative number of ACEs had higher odds of lifetime illicit drug use (adjusted OR = 2.73, 95% CI = 1.25–5.96), riding with a drunk driver during their lifetime (adjusted OR = 2.44, 95% CI = 1.12–5.33), suicidal ideation before the age of 18 (adjusted OR = 28.49, 95% CI = 3.81–212.87), and suicidal ideation in last 12 months (adjusted OR = 5.39, 95% CI = 1.58–18.36) (see Table 3). 

Linear regression was used to predict the psychological well-being of the students from their total ACE score. Total ACE scores explained a significant amount of variance in psychological well-being, *F*(3, 390) = 15.64, *p* < 0.001, *R*^2^ = 0.12. The regression coefficient (*B* = −3.94, 95% CI [−5.08; −2.80]) indicated that an increase in one ACE corresponded, on average, to a decrease in the psychological well-being score by 3.94 points (results not shown).

## 4. Discussion

This study analysed the associations between ACEs, health risk behaviours, and psychological well-being among a convenience cross-sectional sample of university students in Lithuania. Our findings revealed a high proportion of any ACE exposure among our study sample (91.3%) and of polyvictimisation (almost half of our sample experienced four or more ACEs). Results from the present study indicated an increased risk for lifetime drug use, riding with a drunk driver, and suicidal ideation among students with four or more ACE experiences, compared with those who did not experience ACEs. Further, an increased number of ACEs was associated with decreased psychological well-being. Our findings confirm results from prior research indicating the association between ACEs and adverse health outcomes while expanding the research evidence for a convenience sample of Lithuanian university students. 

In this study sample, the most commonly reported traumatic experiences were emotional abuse, emotional neglect, and physical abuse. Our results are generally consistent with other studies, especially regarding emotional abuse [19,45,46]. As for the cumulative number of ACEs, almost half of the study sample (48.9%) experienced four or more ACEs, which is higher than what has been found in other studies [1,2,39,47]. Some of the limitations of ACE measurement across studies pertain to inconsistencies in the inclusion (or not) of some types of household dysfunctions, as well as differences in construct definition and variable assessment [41]. In this study, we coded childhood emotional and physical abuse if participants mentioned experiencing these types of abuse at least once (e.g., [41]), which may explain the higher proportion of these types of abuse among our study sample when compared with other studies that used a higher cutoff point [1].

Students in our sample reported their first substance use experiences before the age of 18, with alcohol having the earliest mean age of initiation. Previous studies showed that experimentation with alcohol, tobacco, and illicit drugs often starts in adolescence [44,48]. Similar to another study [22], we found that ACEs were not statistically significantly associated with alcohol use in the study sample. In line with the findings of Assini-Meytin et al. [22], a large proportion of participants reported alcohol use (in their lifetime, past 12 months, and past 30 days), which may indicate a normative (or common) behaviour among individuals in their early 20s, regardless of ACE risks.

Consistent with other studies, findings from adjusted regression models showed statistically significant associations between the cumulative number of ACEs and lifetime drug use [18,23] and suicidal ideation [22,49]. Notably, Lithuania has substantially higher suicide rates than other European countries [50]. It may be that a history of childhood polyvictimisation among Lithuanian adults contributes to the disproportionate rate of suicide, compared with other European countries. Additional studies, however, are needed to explore the impact of ACEs on suicide rates disparities, as well as, protective factors for suicidal behaviour among Lithuanian university students such as crisis intervention and suicide prevention programs. 

In the present study, almost half of the participants (43.3%) reported having travelled with a driver impaired by alcohol at least once, and among those who had driver’s licences, 16.9% reported driving a car after alcohol consumption. These results are similar to previous findings based on Lithuanian university students [44]. Our findings also showed a statistically significant association between the cumulative number of ACEs and riding with a drunk driver. Students’ attitudes to alcohol use while driving are related to the norms and behaviours of their families and peers [30]. As noted by Nazif-Muñoz and Blank-Gomel [32], university students have more opportunities to be passengers of alcohol-intoxicated drivers because they are exposed to a risk-oriented student culture. The lack of a statistically significant association between ACEs and drunk driving is likely based on a few factors. First, only two out of three students in our sample had a driver’s licence, which may be explained by participants’ young age (mean 21) and the fact that a driver’s licence can be obtained in Lithuania starting from age 18. Second, there is a zero-tolerance drunk driving law for new drivers (i.e., those with less than two years of driver experience), with harsh penalties for breaking it, which may further explain the low proportion of impaired driving in our sample and the lack of statistically significant association with ACEs.

Consistent with findings from previous research [51], our results showed that an increase in ACE scores was associated with lower levels of psychological well-being. In a study with a longitudinal sample of US college students, participants’ psychological well-being was significantly reduced upon college entry [52]. Experiencing multiple traumatic events in childhood may further amplify college students’ vulnerabilities to experiencing lower psychological well-being in their adaptation to college. Further studies that examine the influence of ACEs in college students’ psychological well-being trajectories are warranted. 

It is important to mention several limitations of the current study. First, the study was based on a convenience sample of university students; therefore, findings cannot be generalised to all Lithuanian young adults. In the 2019 academic year, 38% of youth aged 20–24 years were pursuing higher education [53]. It may be that those who experienced greater disadvantages in their childhood did not continue their education beyond high school and were not included in the study sample. Similarly, our results are limited in their generalisability to the entire Lithuanian university student population, particularly males. Therefore, future research would benefit from the inclusion of a more gender-balanced student sample with a broader representation of study programs. Second, our study was based on cross-sectional data with measures informed by participants’ retrospective self-reports, which may generate recall bias. Although recall bias is a commonly reported concern in ACE studies, bias was likely reduced by the use of dichotomised responses (yes/no) about experiences of early life adversity [7]. Third, we expanded the original ACE measures by adding important types of childhood traumatic experiences derived from the literature and relevant to the life of Lithuanian youth (e.g., parental death and parental migration); however, other types of possible traumatic experiences were not included in this study (e.g., bullying and peer violence). Consequently, variations in ACE assessment make a comparison of findings between studies difficult.

Another limitation is that data were collected during the first wave of the COVID-19 pandemic, which may have influenced participants’ reports of risky behaviours and psychological well-being. However, our study’s cross-sectional design does not allow for a wider extrapolation of how the COVID-19 pandemic might have affected the association between ACEs, risky behaviours, and psychological well-being. Finally, it should be noted that in 2017, the Lithuanian Parliament (Seimas) adopted amendments and supplements to the Law on Fundamentals of Protection of the Rights of the Child by which all forms of violence against children were defined and forbidden. Future research is needed to identify whether those changes in legislation had any impact on the various forms of child abuse experienced by contemporaneous cohorts of Lithuanian children.

## 5. Implications and Future Directions

Notwithstanding the above limitations, the findings of this study have some important implications. Much attention should be paid to the prevention of traumatic events in childhood given their considerable adverse impact over the life course [54]. Risky health behaviour prevention programs should focus on public-health-related evidence-based approaches that encompass a wide range of interventions, including the development of self-help programs, education, policy changes, and legislation. Furthermore, results from this study have implications for mental health professionals as well as university administrators, as students with higher numbers of traumatic experiences may need greater levels of support and services, particularly during difficult times such as the COVID-19 pandemic.

## 6. Conclusions

Our study provides unique insights into the types and the cumulative number of traumatic events experienced by a convenience sample of Lithuanian university students. The high proportion of students who experienced four or more forms of traumatic events in childhood (almost half of our sample) and its association with lifetime drug use, riding with a drunk driver, suicide ideation, and reduced psychological well-being highlight the importance of making prevention programs and psychological resources accessible to Lithuanian university students. The challenges associated with the transition to adulthood and adjustment to college [53] might amplify the adverse effects of ACEs among university students and further highlight the need for preventive intervention and psychological support among this population.

## Figures and Tables

**Table 1:** Participant demographic characteristics and descriptive statistics of adverse childhood experiences (ACEs).

	Overall (*n* = 393)	
	*n*	%
Age (mean, *SD*)	393	21.07 ± 1.53
Gender		
Female	317	80.7
Male	76	19.3
Study program		
Social sciences and humanities	305	80.5
Other	88	19.5
Mother’s education		
Lower secondary education or less	20	5.1
Upper secondary education	62	15.8
Higher education (colleges)	97	24.7
Higher education (universities)	203	51.7
Father’s education		
Lower secondary education or less	28	7.1
Upper secondary education	91	23.2
Higher education (colleges)	101	25.7
Higher education (universities)	145	36.9
Living situation		
With parents	135	34.4
Dormitory	95	24.2
Rented apartment (alone or with others)	130	33.1
Other	33	8.4
Possession of driver’s licence	255	64.9
ACEs type		
Emotional abuse	269	68.4
Physical abuse	192	48.9
Sexual abuse	42	10.7
Substance abuse in household	143	36.4
Incarceration in household	11	2.8
Mental illness in household	139	35.4
Witnessing domestic violence	123	31.3
Parental separation or divorce	112	28.5
Parental migration	61	15.5
Deceased parent	25	6.4
Placement in foster home	3	0.8
Emotional neglect	255	64.9
Neglect	53	13.5
ACEs score (mean, *SD*)		3.63 ± 2.37
ACEs score categories		
0	34	8.7
1	55	14.0
2–3	112	28.5
≥4	192	48.9

Primary and lower secondary education, 10 grades; upper secondary education, 12 grades. *SD*, standard deviation.

**Table 2:** Health risk behaviour outcomes and psychological well-being scores in the study sample (*n* = 393) and in four categories of exposure of adverse childhood experiences (ACEs).

Study Variables	Total (*n* = 393)		ACEs Score Categories							
			0		1		2–3		≥4	
	*n*	%	*n*	%	*n*	%	*n*	%	*n*	%
Smoking										
Ever	185	47.1	16	47.1	21	38.2	48	42.9	100	52.1
Age at the first time trying smoking (mean)	185	16.9	16	16.7	21	17.2	48	16.9	100	16.8
12 months	174	44.3	15	44.1	19	34.5	47	42.0	93	48.4
30 days	148	37.7	14	41.2	15	27.3	38	33.9	81	42.2
Alcohol consumption										
Ever	362	92.1	29	85.3	49	89.1	104	92.3	180	93.8
Age at the first time trying alcohol (mean)	362	16.3	29	16.6	49	16.4	104	16.4	180	16.3
12 months	350	89.1	29	85.3	47	85.5	103	92.0	171	89.1
30 days	282	71.8	24	70.6	38	69.1	85	75.9	135	70.3
Illicit drug abuse										
Ever ^¥^	186	47.3	10	29.4	18	32.7	52	46.4	106	55.2
Age at the first time trying drugs (mean)	186	17.8	10	17.6	18	18.3	52	17.7	106	17.8
12 months	104	26.5	8	23.5	7	12.7	31	27.7	58	30.2
30 days	43	10.9	4	11.8	2	3.6	8	7.1	29	15.1
Prescribed drug abuse										
Ever	24	6.1	1	2.9	2	3.6	7	6.3	14	7.3
12 months	9	2.3	0	0	0	0	1	0.9	8	4.2
30 days	3	0.8	0	0	0	0	0	0	3	1.6
Sexual intercourse										
Ever	282	71.8	25	73.5	33	60.0	79	70.5	144	75.0
Age at the first time having sex (mean)	282	17.9	25	18.0	33	18.2	79	18.0	144	17.6
12 months	258	65.6	23	67.6	29	52.7	76	67.9	130	67.7
Partners (≥2) in 12 months	53	13.5	1	2.9	7	12.7	13	11.6	32	16.7
Driving after alcohol consumption	43	10.9	4	11.8	5	9.1	10	8.9	24	12.5
Riding with a drunk driver										
Ever ^¥^	170	43.3	11	32.4	20	36.4	38	33.9	101	52.6
12 months	59	15.0	7	20.6	8	14.5	12	10.7	32	16.7
30 days	18	4.6	2	5.9	3	5.5	3	2.7	10	5.2
Suicidal ideation										
Before age18 ^¥^	133	33.8	1	2.9	11	20.0	31	27.7	90	46.9
12 months	109	27.7	3	8.8	13	23.6	28	25.0	65	33.9
LPGS-P total (mean) ^¥^	393	116.8	34	133.6	55	131.5	112	119.8	192	107.9

LPGS-P total, psychological well-being total score. ^¥^ mean differences between ACEs score categories that are significant at level of *p* < 0.008.

**Table 3:** Multivariable logistic regression model’s estimation of the association between health risk behaviours and ACE score categories.

Outcome	ACEs Score Categories		
	1	2–3	≥4
	Adjusted OR (95% CI)		
Illicit drug abuse			
Ever	1.10 (0.44, 2.76)	1.91 (0.84, 4.32)	2.73 (1.25, 5.96) *
Riding with a drunk driver			
Ever	1.30 (0.52, 3.24)	1.12 (0.49, 2.57)	2.44 (1.12, 5.33) *
Suicidal ideation			
Before age 18	7.78 (0.95, 63.48) ^	12.43 (1.63, 95.06) *	28.49 (3.81, 212.87) **
12 months	3.17 (0.83, 12.14) ^	3.48 (0.98, 12.29) ^	5.39 (1.58, 18.36) **

Model incorporates ACE score categories. Referent group, 0 ACEs; OR, odds ratio; CI, confidence interval. All models were adjusted for age and biological sex. Logistic regression models met the following parameters: Wald statistics *p* < 0.05; classification > 50%; Cook’s distance ≤ 1 and Hosmer–Lemeshow test *p* ≥ 0.05. ** = *p* < 0.008; * = *p* < 0.05; ^ = *p* < 0.10.

## Data Availability

The data presented in this study are available on a reasonable request from the corresponding author.

## References

[1] Felitti V.J., Anda R.F., Nordenberg D., Williamson D.F., Spitz A.M., Edwards V., Koss M.P., Marks J.S. (1998). Relationship of Childhood Abuse and Household Dysfunction to Many of the Leading Causes of Death in Adults. Am. J. Prev. Med..

[2] Kappel R.H., Livingston M.D., Patel S.N., Villaveces A., Massetti G.M. (2021). Prevalence of Adverse Childhood Experiences (ACEs) and Associated Health Risks and Risk Behaviors among Young Women and Men in Honduras. Child Abuse Negl..

[3] Larkin H., Shields J.J., Anda R.F. (2012). The Health and Social Consequences of Adverse Childhood Experiences (ACE) across the Lifespan: An Introduction to Prevention and Intervention in the Community. J. Prev. Interv. Community.

[4] Felitti V.J., Anda R.F., Nordenberg D., Williamson D.F., Spitz A.M., Edwards V., Koss M.P., Marks J.S. (2019). Relationship of Childhood Abuse and Household Dysfunction to Many of the Leading Causes of Death in Adults: The Adverse Childhood Experiences (ACE) Study. Am. J. Prev. Med..

[5] McLaughlin K.A., Green J.G., Gruber M.J., Sampson N.A., Zaslavsky A.M., Kessler R.C. (2012). Childhood Adversities and First Onset of Psychiatric Disorders in a National Sample of Adolescents. Arch. Gen. Psychiatry.

[6] Nelson C.A., Bhutta Z.A., Burke Harris N., Danese A., Samara M. (2020). Adversity in Childhood Is Linked to Mental and Physical Health throughout Life. BMJ.

[7] Nurius P.S., Green S., Logan-Greene P., Borja S. (2015). Life Course Pathways of Adverse Childhood Experiences toward Adult Psychological Well-Being: A Stress Process Analysis. Child Abuse Negl..

[8] Campbell J.A., Walker R.J., Egede L.E. (2016). Associations between Adverse Childhood Experiences, High-Risk Behaviors, and Morbidity in Adulthood. Am. J. Prev. Med..

[9] Dube S.R., Williamson D.F., Thompson T., Felitti V.J., Anda R.F. (2004). Assessing the Reliability of Retrospective Reports of Adverse Childhood Experiences among Adult HMO Members Attending a Primary Care Clinic. Child Abuse Negl..

[10] Ford K., Bellis M.A., Hughes K., Barton E.R., Newbury A. (2020). Adverse Childhood Experiences: A Retrospective Study to Understand Their Associations with Lifetime Mental Health Diagnosis, Self-Harm or Suicide Attempt, and Current Low Mental Wellbeing in a Male Welsh Prison Population. Health Justice.

[11] Gilbert L.K., Breiding M.J., Merrick M.T., Thompson W.W., Ford D.C., Dhingra S.S., Parks S.E. (2015). Childhood Adversity and Adult Chronic Disease. Am. J. Prev. Med..

[12] Wang Y.-R., Sun J.-W., Lin P.-Z., Zhang H.-H., Mu G.-X., Cao F.-L. (2019). Suicidality among Young Adults: Unique and Cumulative Roles of 14 Different Adverse Childhood Experiences. Child Abuse Negl..

[13] Figueiredo-Braga M., Dias A., Becker J., Veloso J., Sales L., Lotzin A., Acquarini E., Ardino V., Arnberg F., Böttche M. (2021). The Burden of Adverse Childhood Experiences When Coping and Adjust to COVID-19 Pandemic. Trauma and Mental Health during the Global Pandemic.

[14] Hillis S., Mercy J., Amobi A., Kress H. (2016). Global Prevalence of Past-Year Violence against Children: A Systematic Review and Minimum Estimates. Pediatrics.

[15] Hughes K., Bellis M.A., Hardcastle K.A., Sethi D., Butchart A., Mikton C., Jones L., Dunne M.P. (2017). The Effect of Multiple Adverse Childhood Experiences on Health: A Systematic Review and Meta-Analysis. Lancet Public Health.

[16] Petruccelli K., Davis J., Berman T. (2019). Adverse Childhood Experiences and Associated Health Outcomes: A Systematic Review and Meta-Analysis. Child Abuse Negl..

[17] Sethi D., Bellis M.A., Hughes K., Mitis F., Gilbert R., Galea G. (2013). European Report on Preventing Child Maltreatment.

[18] Bellis M.A., Hughes K., Leckenby N., Jones L., Baban A., Kachaeva M., Povilaitis R., Pudule I., Qirjako G., Ulukol B. (2014). Adverse Childhood Experiences and Associations with Health-Harming Behaviours in Young Adults: Surveys in Eight Eastern European Countries. Bull. World Health Organ..

[19] Giordano F., Caravita S.C.S., Jefferies P. (2020). Social-Ecological Resilience Moderates the Effectiveness of Avoidant Coping in Children Exposed to Adversity: An Exploratory Study in Lithuania. Front. Psychol..

[20] Zelviene P., Daniunaite I., Hafstad G.S., Thoresen S., Truskauskaite-Kuneviciene I., Kazlauskas E. (2020). Patterns of Abuse and Effects on Psychosocial Functioning in Lithuanian Adolescents: A Latent Class Analysis Approach. Child Abuse Negl..

[21] Steptoe A., Fink G. (2007). Health Behaviour and Stress. Encyclopedia of Stress.

[22] Assini-Meytin L.C., Fix R.L., Green K.M., Nair R., Letourneau E.J. (2021). Adverse Childhood Experiences, Mental Health, and Risk Behaviors in Adulthood: Exploring Sex, Racial, and Ethnic Group Differences in a Nationally Representative Sample. J. Child Adolesc. Trauma.

[23] El Mhamdi S., Lemieux A., Bouanene I., Ben Salah A., Nakajima M., Ben Salem K., Al’Absi M. (2017). Gender Differences in Adverse Childhood Experiences, Collective Violence, and the Risk for Addictive Behaviors among University Students in Tunisia. Prev. Med..

[24] Fuller-Thomson E., Baird S.L., Dhrodia R., Brennenstuhl S. (2016). The Association between Adverse Childhood Experiences (ACEs) and Suicide Attempts in a Population-Based Study. Child Care Health Dev..

[25] Lim S.S., Vos T., Flaxman A.D., Danaei G., Shibuya K., Adair-Rohani H., AlMazroa M.A., Amann M., Anderson H.R., Andrews K.G. (2012). A Comparative Risk Assessment of Burden of Disease and Injury Attributable to 67 Risk Factors and Risk Factor Clusters in 21 Regions, 1990–2010: A Systematic Analysis for the Global Burden of Disease Study 2010. Lancet.

[26] Peacock A., Leung J., Larney S., Colledge S., Hickman M., Rehm J., Giovino G.A., West R., Hall W., Griffiths P. (2018). Global Statistics on Alcohol, Tobacco and Illicit Drug Use: 2017 Status Report. Addiction.

[27] Griswold M.G., Fullman N., Hawley C., Arian N., Zimsen S.R.M., Tymeson H.D., Venkateswaran V., Tapp A.D., Forouzanfar M.H., Salama J.S. (2018). Alcohol Use and Burden for 195 Countries and Territories, 1990–2016: A Systematic Analysis for the Global Burden of Disease Study 2016. Lancet.

[28] Organisation for Economic Cooperation and Development (OECD), European Observatory on Health Systems and Policies (2019). Lithuania: Country Health Profile 2019.

[29] European Transport Safety Council (2018). Progress in Reducing Drink Driving in Europe. https://etsc.eu/wp-content/uploads/report_reducing_drink_driving_final.pdf.

[30] Leadbeater B.J., Foran K., Grove-White A. (2008). How Much Can You Drink before Driving? The Influence of Riding with Impaired Adults and Peers on the Driving Behaviors of Urban and Rural Youth. Addiction.

[31] Poulin C., Boudreau B., Asbridge M. (2007). Adolescent Passengers of Drunk Drivers: A Multi-Level Exploration into the Inequities of Risk and Safety. Addiction.

[32] Nazif-Muñoz J.I., Blank-Gomel A. (2017). Passengers at Risk: A Multi-Level Analysis of the Decision to Travel with a Drunk Driver. Addiction.

[33] Gailienė D., Gailienė D. (2015). Suicides in Lithuania: Sociocultural Context. Lithuanian Faces after Transition: Psychological Consequences of Cultural Trauma.

[34] Institute of Hygiene (2016). Lietuvos jaunimo sveikatos būklė. Visuomenės Sveik. Netolygumai.

[35] Lekečinskaitė L., Lesinskienė S. (2017). Vaikų ir jaunimo iki 20 m. savižudybės Lietuvoje 2010–2015 m. [Suicides of Children and Young Adults under 20 in Lithuania in 2010–2015]. Visuomenės Sveik..

[36] Bagdonas A., Pakalniškienė V., Kairys A., Liniauskaitė A. (2013). Lietuvos Gyventojų Psichologinė Gerovė ir Jos Veiksniai [Psychological Well-Being and Its Factors in Lithuania].

[37] Charry C., Goig R., Martínez I. (2020). Psychological Well-Being and Youth Autonomy: Comparative Analysis of Spain and Colombia. Front. Psychol..

[38] Ryff C.D. (2014). Psychological Well-Being Revisited: Advances in the Science and Practice of Eudaimonia. Psychother. Psychosom..

[39] Merrick M.T., Ford D.C., Ports K.A., Guinn A.S., Chen J., Klevens J., Metzler M., Jones C.M., Simon T.R., Daniel V.M. (2019). Vital Signs: Estimated Proportion of Adult Health Problems Attributable to Adverse Childhood Experiences and Implications for Prevention—25 States, 2015–2017. Morb. Mortal. Wkly. Rep..

[40] Huang C.-C., Tan Y., Cheung S.P., Hu H. (2021). Adverse Childhood Experiences and Psychological Well-Being in Chinese College Students: Mediation Effect of Mindfulness. Int. J. Environ. Res. Public Health.

[41] Merians A.N., Baker M.R., Frazier P., Lust K. (2019). Outcomes Related to Adverse Childhood Experiences in College Students: Comparing Latent Class Analysis and Cumulative Risk. Child Abuse Negl..

[42] Browning M.H.E.M., Larson L.R., Sharaievska I., Rigolon A., McAnirlin O., Mullenbach L., Cloutier S., Vu T.M., Thomsen J., Reigner N. (2021). Psychological Impacts from COVID-19 among University Students: Risk Factors across Seven States in the United States. PLoS ONE.

[43] Centers for Disease Control and Prevention About the CDC-Kaiser ACE Study. Violence Prevention Injury Center, CDC. https://www.cdc.gov/violenceprevention/aces/about.html.

[44] Bulotaitė L. (2014). Rizikingas elgesys: Samprata, Paplitimas, Veiksniai. Monografija [Risky Behavior: Concept, Prevalence, Factors. Monograph].

[45] Bertolino D.F., Sanchez T.H., Zlotorzynska M., Sullivan P.S. (2020). Adverse Childhood Experiences and Sexual Health Outcomes and Risk Behaviors among a Nationwide Sample of Men Who Have Sex with Men. Child Abuse Negl..

[46] Finkelhor D., Turner H., Ormrod R., Hamby S.L. (2009). Violence, Abuse, and Crime Exposure in a National Sample of Children and Youth. Pediatrics.

[47] Thomas J.T. (2016). Adverse Childhood Experiences among MSW Students. J. Teach. Soc. Work.

[48] Flanagan E., Bedford D., O’Farrell A., Browne C., Howell F. (2004). Smoking, Alcohol & Illicit Drug Use among Young People in a Health Board Region in 1997 and 2002: A Comparative Study. Ir. Med. J..

[49] Karatekin C. (2018). Adverse Childhood Experiences (ACEs), Stress and Mental Health in College Students. Stress Health.

[50] Eurostat (2020). Almost 8 in 10 Suicides among Men. https://ec.europa.eu/eurostat/web/products-eurostat-news/-/edn-20200910-1.

[51] Mosley-Johnson E., Garacci E., Wagner N., Mendez C., Williams J.S., Egede L.E. (2018). Assessing the Relationship between Adverse Childhood Experiences and Life Satisfaction, Psychological Well-Being, and Social Well-Being: United States Longitudinal Cohort 1995–2014. Qual. Life Res..

[52] Conley C.S., Kirsch A.C., Dickson D.A., Bryant F.B. (2014). Negotiating the Transition to College. Emerg. Adulthood.

[53] Official Statistics Portal (2021). Education, Culture and Sports in Lithuania. https://osp.stat.gov.lt/lietuvos-svietimas-kultura-ir-sportas-2021/aukstasis-mokslas.

[54] Centers for Disease Control and Prevention (2019). Preventing Adverse Childhood Experiences (ACEs): Leveraging the Best Available Evidence.

